# Hormonal Effects of an Enzymatically Hydrolyzed Animal Protein-Based Biostimulant (Pepton) in Water-Stressed Tomato Plants

**DOI:** 10.3389/fpls.2019.00758

**Published:** 2019-06-12

**Authors:** Andrea Casadesús, Javier Polo, Sergi Munné-Bosch

**Affiliations:** ^1^Department of Evolutionary Biology, Ecology and Environmental Sciences, University of Barcelona, Barcelona, Spain; ^2^R&D Department, APC Europe S.L., Granollers, Spain; ^3^Institut de Nutrició i Seguretat Alimentària (INSA), University of Barcelona, Barcelona, Spain

**Keywords:** auxin, biostimulants, cytokinins, defenses, jasmonic acid, tocopherols, tomato

## Abstract

Biostimulants may promote growth or alleviate the negative effects of abiotic stress on plant growth eventually resulting in enhanced yields. We examined the mechanism of action of an enzymatically hydrolyzed animal protein-based biostimulant (Pepton), which has previously been shown to benefit growth and yield in several horticultural crops, particularly under stressful conditions. Tomato plants were exposed to well-watered and water-stressed conditions in a greenhouse and the hormonal profiling of leaves was measured during and after the application of Pepton. Results showed that the Pepton application benefited antioxidant protection and exerted a major hormonal effect in leaves of water-stressed tomatoes by increasing the endogenous content of indole-3-acetic acid (auxin), *trans*-zeatin (cytokinin), and jasmonic acid. The enhanced jasmonic acid content may have contributed to an increased production of tocochromanols because plastochromanol-8 concentration per unit of chlorophyll was higher in Pepton-treated plants compared to controls. In conclusion, the tested Pepton application may exert a positive effect on hormonal balance and the antioxidant system of plants under water stress in an economically important crop, such as tomato plants.

## Introduction

Biostimulants are re-emerging as important tools to improve yields and alleviate the negative effects of stress in horticultural crops (du Jardin, 2015). Among the different categories of biostimulants, enzymatically hydrolyzed animal protein-based biostimulants represent a cost-effective approach to alleviate the negative effects of different types of stress in horticultural crops (Polo et al., 2006; Phelan et al., 2009; Colla et al., 2014, 2017; Polo and Mata, 2018). Pepton 85/16^®^ (Pepton) is a natural biostimulant product obtained by proprietary enzymatic hydrolysis of animal protein available in micro-granular form and highly soluble in water (APC Europe S.L., Spain). Pepton has demonstrated beneficial effects on commercial crops, especially under abiotic stress conditions. Pepton reduced the negative effects caused by intense cold or heat episodes in lettuce and, at the highest inclusion level tested, Pepton completely reversed the negative impact of the cold or heat induced thermal stress (Polo et al., 2006). Similarly, in strawberry plants stressed by being transplanted and subjected to conditions of intense cold ambient temperatures, Pepton application accelerated newly formed roots, flowering, and production of fruit (Marfà et al., 2009). Recently, in a study using gold cherry tomatoes grown under mild stress ambient field conditions, Pepton application at different inclusion levels (from 2 to 4 kg/ha) resulted in a linear improvement of all vegetative growth parameters and yield was 27% higher compared to the control treatment (Polo and Mata, 2018).

Phytohormones are crucial to vegetative growth regulation. Their cross-talk is responsible for the coordination of several plant growth and developmental processes (Davies, 2010). They coordinate most plant developmental processes in response to internal and external factors, with auxins, CKs, and GAs generally promoting vegetative growth (Wolters and Jürgens, 2009; Davies, 2010). Auxin bioactive form (IAA) promotes vegetative growth of the whole plant through polar transport mediated by PIN proteins that result in a basipetal auxin gradient that regulates cell expansion, cell differentiation, morphogenesis and organogenesis (Pacifici et al., 2015). CKs are adenosine- and isoprenoid-derived compounds and the most common bioactive form, *t*Z, plays an essential role in modulation of cell division and the establishment of source-sink relations within the plant (Mok and Mok, 2001). Bioactive GAs (e.g., GA_1_ and GA_3_) are essential compounds in the regulation of plant size, play a key role in the regulation of cell expansion, and regulate key developmental processes in the plant life cycle, such as seed germination and flower development (Pacifici et al., 2015). Major stress-related phytohormones include ABA, ethylene, jasmonates, and salicylates (Wolters and Jürgens, 2009). ABA, which is derived from carotenoids (violaxanthin or neoxanthin), mediates the response to environmental stress stimuli, i.e., drought stress or salt stress, to typically close stomata, accumulate compatible osmolytes and modulate the expression of stress-related genes that will ultimately reduce vegetative growth and provide tolerance to desiccation (Wolters and Jürgens, 2009; Pacifici et al., 2015). JA is an oxylipin derived from chloroplasts-located fatty acids, such as linolenic acid, with a major role in the coordination of the defense against biotic stress, such as herbivore attack or necrotrophic pathogen infection (Wang and Wu, 2013). Also, JA has been associated with abiotic stress resistance to ultraviolet radiation or ozone, and with different aspects of development (Huang et al., 2017). SA is a phenolic acid derived from chorismate that is also involved in biotic stress response (mainly biotrophic pathogens) retarding plant growth and inducing pathogen-related genes, mainly in interaction with ABA and JA (Ku et al., 2018).

Phytohormones have been previously linked with biostimulants in horticultural crops. Several seaweed and plant extracts have been shown to possess CKs-like and auxin-like activities (Stirk et al., 2014), and also hormonal activity due to the presence of GAs, brassinosteroids, ABA, SA, and/or JA (Stirk et al., 2014; Elzaawely et al., 2016; Kulkarni et al., 2019). Higher endogenous auxin and GAs contents in plants have been reported after biostimulant application using humic substances (Aremu et al., 2015). Abbas (2013) observed higher endogenous ABA contents in *Vicia faba* treated with microbial biostimulants, seaweed extracts, and humic substances. Furthermore, Desoky et al. (2018) showed higher auxins, GAs and CKs contents in sorghum plants under salt stress conditions that were treated with humic substances or vegetable extracts as biostimulants. Despite several examples of hormone-like effects for biostimulants in horticultural crops (Cohen and Bandurski, 1982; Philipson, 1985; Parrado et al., 2008), the mechanism of action of hydrolyzed animal peptides as biostimulants remains largely unknown. Hydrolyzed animal peptides contain several amino acids, in particular aromatic amino acids such as tryptophan and phenylalanine which are precursors for the synthesis of auxin, which has been suggested as a key to explain their action (Dai et al., 2013; Zhao, 2014). Furthermore, glutamic acid, glycine and, to a lesser extent, alanine and arginine have been considered fundamental metabolites in the process of chlorophyll synthesis (Von Wettstein et al., 1995). However, research is needed to better understand possible mechanisms of action of these products in economically interesting crops under stress conditions. Therefore, the aim of this work was to establish a mechanism of action for an enzymatically hydrolyzed animal protein-based biostimulant (Pepton), which has previously been shown to promote growth and yield in several horticultural crops, particularly under stressful conditions.

## Materials and Methods

### Growth Conditions, Treatments, and Samplings

Tomatoes were chosen as a model crop for studying actions of biostimulant products because they are a high value crop produced worldwide with a relatively short growing period and less expensive to use in research studies compared with other crops. In addition, there is considerable information on the variation of physiological components of tomatoes associated with stress conditions. Seeds of tomato (*Lycopersicon esculentum*, var. “Ailsa Craig”), which were obtained from the Experimental Field Facilities of the University of Barcelona, were sown in 1 dm^3^ pots under long-day conditions in a growth chamber (12 h light/12 h dark) on March 23, 2018. After 1 month of growth, seedlings were transplanted to 3 dm^3^ pots and placed in a greenhouse with a distance between pots of 20 cm. Four treatments were established, including well-watered plants without Pepton, well-watered plants with Pepton, water-stressed plants without Pepton and water-stressed plants with Pepton. The well-watered condition was developed with daily, constant irrigation of plants with Hoagland nutritive solution maintaining whole pot field capacity, according to their evapotranspirative demand. Water deficit conditions were established by irrigating plants with 0.5 dm^3^ of Hoagland nutritive solution every 2 days during the first 6 weeks, and later with 1 dm^3^ every 2 days to the end of the treatment period, responding to the increase of evapotranspirative demand as the season progressed toward mid-summer (Supplementary Figure 1). Pepton was applied by ferti-irrigation once every 2 weeks at a dose equivalent of 4 Kg/ha, that is 200 mg of Pepton dissolved in 0.5 dm^3^ of irrigation water, which corresponds to the highest level of supplier recommendation for this crop. Applications were performed 1 h before sunset. Leaf samples were collected May 10 (week 0, start of the experiment), May 25 (week 2), June 8 (week 4), July 5 (week 8), and August 2 (week 12) at predawn (1 h before sunrise). Two young, fully developed leaves were sampled. One leaf was immediately frozen in liquid nitrogen and stored at −80°C for hormonal profile analyses. The other leaf was used to determine the *F*_v_/*F*_m_ *in situ* with the mini-PAM II (Photosynthesis Yield Analyser, Walz, Germany). Then the leaf blade was cut in two symmetric parts. One part was used to determine the RWC [calculated as 100 × (FW - DW)/TW - DW), where FW is the fresh weight, TW is the turgid weight after 24 h immersed in water, and DW is the dry weight after drying in the oven at 80°C]. The other part was immediately frozen in liquid nitrogen and stored at −80°C to perform analyses of tocochromanols and photosynthetic pigments.

**FIGURE 1:**
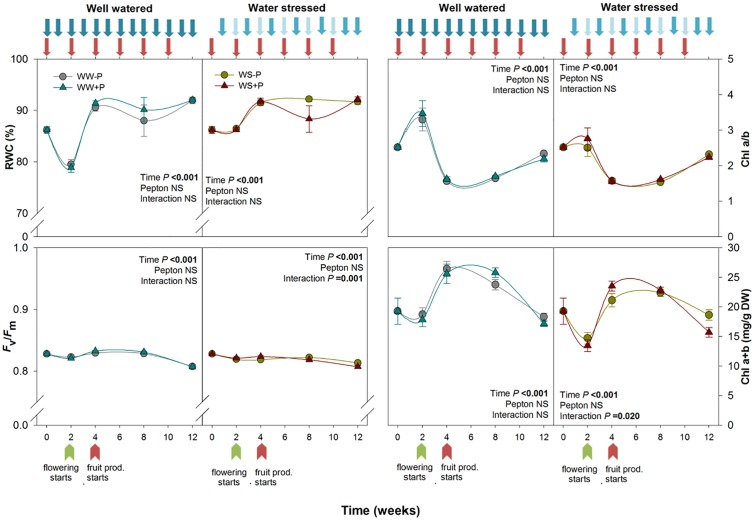
Variations in stress indicators [including the relative water content (RWC), photosystem II maximum quantum yield efficiency (*F*_v_/*F*_m_), chlorophyll *a*/*b* ratio, and total chlorophyll content] in Pepton-treated tomato plants compared to controls under well-watered or water-stressed conditions. Data are the means ± SE of *n* = 10 individuals. Results of statistics (two-way ANOVA with Tukey HSD *post hoc* test) are shown in the inlets. Arrows represent days of watering and Pepton application.

### Pepton Composition

Pepton is an enzymatically hydrolyzed animal protein product that contains L-α amino acids (84.8%), free amino acids (16.5%), organic-nitrogen (12.0%), iron (3000 ppm), and potassium (4.0%). The average molecular weight distribution of Pepton is around 2,000–3,000 Da, from which 66% of the peptides are considered short-chain (with less than 50 amino acids per chain) and 16% are considered long-chain peptides (>50 amino acids). A complete chemical composition of Pepton is reported by Polo and Mata (2018) and is available as Supplementary Table 1.

### Hormonal Profiling

Hormonal profiling was performed by liquid chromatography coupled to electrospray ionization tandem mass spectrometry (UHPLC/ESI-MS/MS) as described by Müller and Munné-Bosch (2011). ABA, GA_1_ and GA_3_, IAA, JA, and SA were analyzed using negative ion mode and the CKs, IPA, *t*Z, and *t*ZR using positive ion mode. Extracts were performed using 100 mg of well powdered fresh leaf with a mixture of methanol and acetic acid (99:1, v/v) as a solvent. Deuterium-labeled plant hormones were added to the extract and 250 mm^3^ of the final mixture was vortexed and ultra-sonicated for 30 min (Branson 2510 ultrasonic cleaner, Bransonic, Danbury, CT, United States). Then the extract was vortexed again and centrifuged for 10 min at 4°C and 200 g. The supernatant was collected, filtered using a 0.22 μm PTFE filter (Waters, Milford, MA, United States) and injected into the HPLC/ESI-MS/MS system.

### Tocochromanols Analyses

Tocochromanols analyses included the determination of α, β, γ, δ-tocopherol, α, β, γ, δ-tocotrienol and plastochromanol-8. Analyses were performed by high-performance liquid chromatography (HPLC) using methanolic extracts as described by Cela et al. (2011). 100 mg of well powdered fresh leaf were used for the extract, and 300 mm^3^ was filtered using a 0.22 μm PTFE filter and injected into the HPLC system (consisting of a Waters 600 controller pump, Waters 714 plus auto-sampler and Jasco FP-1520 fluorescence detector). The mobile phase was a mixture of *n*-hexane and 1,4-dioxane (95.5:4.5, v/v) at a flow rate of 0.7 cm^3^/min. Tocopherol homologues were separated on a normal-phase column (Inertsil 100A, 5 μm, 30 × 250 mm, GL Sciences Inc., Japan). Fluorescence detection was at an excitation wavelength of 295 nm and emission at 330 nm. Standards of α, β, γ, δ-tocopherol, α, β, γ, δ-tocotrienol and plastochromanol-8 (Sigma-Aldrich) were used for calibration.

### Photosynthetic Pigments Analyses

Photosynthetic pigments, including chlorophyll *a*, *b* and total carotenoids, were analyzed using UV/Visible spectroscopy of double beam as described by Oliván and Munné-Bosch (2010). Methanolic extracts were prepared using 100 mg of well powdered fresh leaf and were diluted 1:5 (v/v). The absorbance was read at 470, 653, and 666 nm using a CE Aquarius UV/Visible Spectrophotometer (Cecyl Instruments Ltd., Cambridge, United Kingdom) and pigment concentrations were obtained using the equations developed by Lichtenthaler (1987).

### Statistical Analyses

To determine the effect of “Pepton” and “time,” multifactorial analyses of two fixed factors using two-way ANOVA and Tukey HSD *post hoc* test were performed. Differences were considered significant when *P* ≤ 0.05. Normality and homoscedasticity of residues were checked as described by Zuur et al. (2009). Principal Component Analysis (PCA) was performed using all variables measured, previously standardized in a range value from 0 to 1 to avoid effects of the magnitude of values. All statistical tests were performed using R statistical software (R Foundation for Statistical Computing, Vienna, Austria).

## Results and Discussion

Protein hydrolysates have been demonstrated to exert beneficial effects in alleviating stress in horticultural crops (du Jardin, 2015). The signaling role of amino acids and peptides and their effect on hormone profiling is suggested to play a key role in improving plant performance (Yakhin et al., 2017). Positive effects of protein hydrolysates have been reported under a different type of stress, such as drought, salinity, heavy metals (Phelan et al., 2009; Colla et al., 2017) and thermal stress (Polo et al., 2006; Kauffman et al., 2007; Marfà et al., 2009) but products of animal origin remain poorly studied. Understanding mechanisms of action of animal-derived biostimulants will undoubtedly contribute to better management of horticultural crops under non-favorable conditions which are increasingly important in the current global climate change scenario (Le Quéré et al., 2018; Nogueira et al., 2019). In the present study, the average temperature in the greenhouse was 26.3°C. The minimum and maximum mean daily temperatures occurred at the start and end of the experiment (18.5 and 30.9°C during May 13 and August 2, respectively). The mean daily relative humidity during the study was 58.2%, with a maximum of 75.8% during week 4 and a minimum of 46.0% near the end of the experiment (Supplementary Figure 1). Both well-watered and water-stressed plants showed RWC above 80% and *F*_v_/*F*_m_ values above 0.75 throughout the study. The water deficiency in water-stressed plants was mild compared to irrigated plants because it did not cause differential effects on RWC of fully developed young leaves and the youngest leaves recovered first, but it did cause strong reductions in plant growth. Plant biomass was reduced in water-stressed plants compared to well-watered ones and Pepton slightly alleviated the water-stressed phenotype (Supplementary Figure 2). Chlorophyll *a*/*b* ratio ranged between 1.5 and 4 for well-watered plants and between 1.5 and 3 for water-stressed plants, respectively. Total chlorophyll was around 20–25 mg/g DW in well-watered plants, and between 13 and 23 mg/g DW in water-stressed plants. Pepton had no effect on photosynthetic pigments (Figure 1).

**FIGURE 2:**
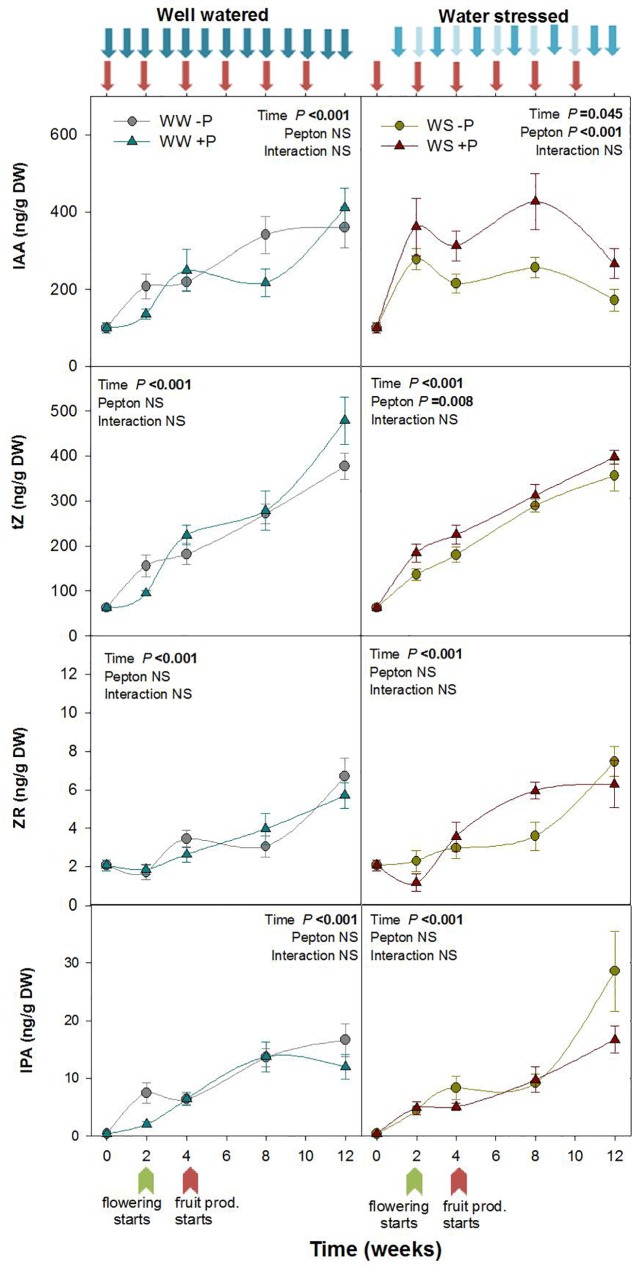
Variations in the endogenous content of auxin and cytokinins in Pepton-treated tomato plants compared to controls under well-watered or water-stressed conditions. Data are the means ± SE of *n* = 10 individuals. Results of statistics (two-way ANOVA with Tukey HSD *post hoc* test) are shown in the inlets. Arrows represent days of watering and Pepton application. DW, dry weight.

### Pepton Increased Growth-Related Phytohormones Under Stressful Conditions

Recent studies suggest that biostimulants based on protein hydrolysates improve crop performance by stimulating carbon, nitrogen and hormonal metabolism of plants (Colla et al., 2017). Several studies have reviewed hormone-like activity of protein hydrolysates of animal origin in crops (Phelan et al., 2009; Lachhab et al., 2014; Colla et al., 2017). However, in the present study we directly evaluated endogenous changes in hormone profile produced by biostimulant application to better understand how biostimulants can improve crop development under stressful environments. Pepton treatment revealed a significant effect on the contents of major growth-related phytohormones, but only under water stress conditions. Under water-stressed conditions, auxin IAA was higher in Pepton-treated plants compared to controls at all sampling times of the study (*P* < 0.001; Figure 2). The highest magnitude of differences was observed during weeks 4 and 8 with Pepton-treated plants having 44 to 66% higher auxin IAA than the controls (Figure 2). An enhanced growth due to the auxin-like activity has also been previously observed (Colla et al., 2014; Ugolini et al., 2015; Elzaawely et al., 2016; Desoky et al., 2018), in part related to an activation of expansions and other auxin-responsive genes (Ertani et al., 2017), using protein hydrolysate products of plant origin. Additionally, the enhanced production of auxin may also in part be related to the high levels of phenylalanine (5.93%) and moderate levels of Trp (1.25%) in Pepton, two well-known precursors of auxin synthesis.

The active cytokinin (*t*Z) concentration was also higher in Pepton-treated plants compared to controls under water stress conditions (*P* = 0.008) with 25–30% higher tZ observed during weeks 2 and 4. The concentration of non-active CKs, including the ribosides ZR and IPA, did not differ between Pepton-treated plants and controls under water stress or irrigated conditions (Figure 2). Elzaawely et al. (2016) and Desoky et al. (2018) also observed higher CKs in leaves using plant extracts as a biostimulant, and the same results were observed by Aremu et al. (2016) and Kulkarni et al. (2019) using seaweed extracts as a biostimulant.

**FIGURE 3:**
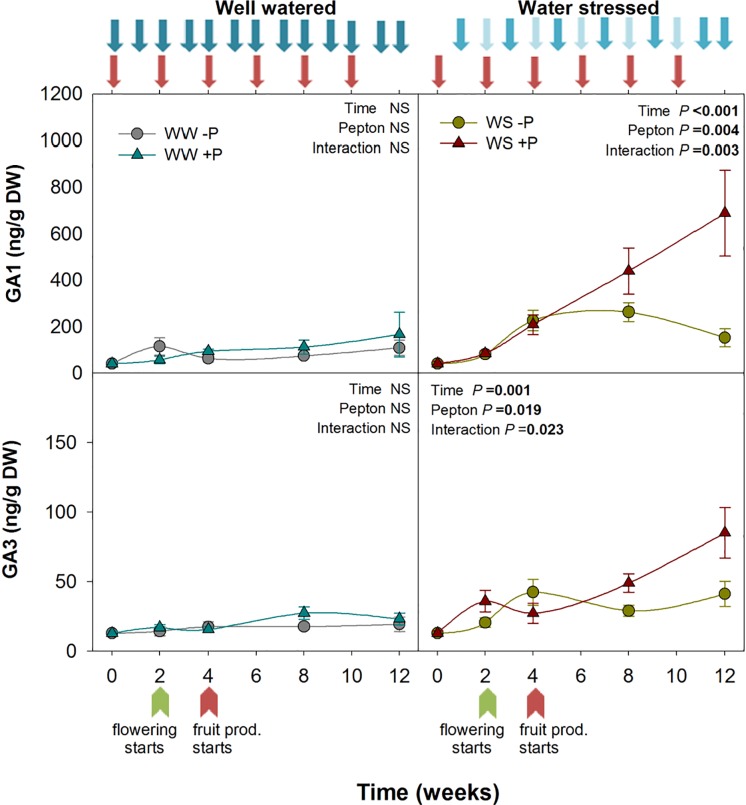
Variations in the endogenous content of gibberellins in Pepton-treated tomato plants compared to controls under well-watered or water-stressed conditions. Data are the means ± SE of *n* = 10 individuals. Results of statistics (two-way ANOVA with Tukey HSD *post hoc* test) are shown in the inlets. Arrows represent days of watering and Pepton application. DW, dry weight.

Bioactive GAs are involved in several developmental processes of plants, i.e., plant size or flowering. Pepton treatment also led to significantly higher GA_1_ and GA_3_ under water stress conditions (*P* = 0.004 and *P* = 0.019, respectively). GA_1_ contents increased progressively from the start to the end of the study in Pepton-treated plants, while this increase did not occur in non-treated plants. At week 12, GA_1_ and GA_3_ contents of Pepton-treated plants were six and threefolds higher, respectively, compared to non-treated plants. GAs did not show the same pattern as other growth related phytohormones under well-watered conditions. Under non-stressful conditions, GA_1_ and GA_3_ concentrations remained between 40 and 100 ng/g DW and between 13 and 20 ng/g DW for GA_1_ and GA_3_, respectively, in both Pepton and non-treated plants (Figure 3). Other studies using plant extracts as biostimulants have confirmed GAs-like activity of these products with greater shoot elongation (Colla et al., 2014; Ertani et al., 2017; Desoky et al., 2018), and flowering (Elzaawely et al., 2016). Most of these studies attributed this effect to the presence of GAs in the plant extract.

### Pepton Improved Defense Response Under Water-Stress Conditions

**FIGURE 4:**
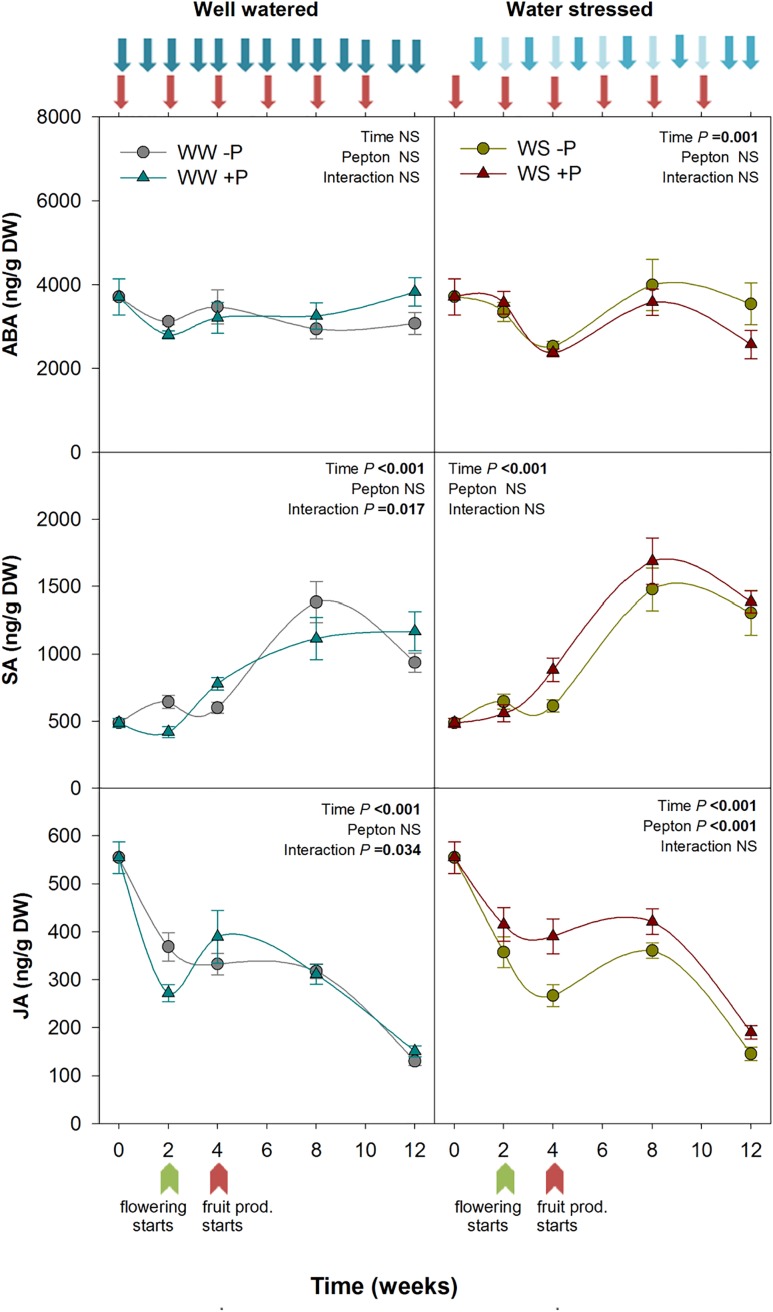
Variations in the endogenous content of abscisic acid, salicylic acid, and jasmonic acid in Pepton-treated tomato plants compared to controls under well-watered or water-stressed conditions. Data are the means ± SE of *n* = 10 individuals. Results of statistics (two-way ANOVA with Tukey HSD *post hoc* test) are shown in the inlets. Arrows represent days of watering and Pepton application. DW, dry weight.

Stress related phytohormones are crucial to improve plant performance under non-favorable conditions. JAs are closely related to biotic stress (Wolters and Jürgens, 2009) but they are also involved in abiotic stress response such as drought (Jubany-Marí et al., 2010; Riemann et al., 2015; Ahmad et al., 2016). Using protein hydrolysates of plant origin, Ertani et al. (2017) demonstrated higher expression of several ethylene/JA/ABA responsive genes including wound-induced proteins and heat shock proteins which are crucial in both biotic and abiotic stress response. In the present study, we found a significant effect of Pepton application on stress-related phytohormones under stressful conditions, specifically for JA contents (*P* < 0.001; Figure 4). Pepton-treated plants under water stress conditions maintained higher JA values than non-treated plants throughout the study. We observed that JA concentrations markedly declined from the start to the end of the study. At week 0, JA was 555 ng/g DW, while by week 4 it had decreased 52% in non-treated plants but only 30% in Pepton-treated plants. By the end of the study, JA in Pepton-treated plants was 66% lower than at the start, but 73% lower in non-treated plants (Figure 4). Overall, the enhanced JA content observed in Pepton-treated plants may lead to greater expression of stress responsive genes. The JA biosynthetic pathway has been thoroughly studied in tomato plants. Because it is derived from fatty acids of the cell membrane (Ahmad et al., 2016), the enhanced JA synthesis observed in the present study appears to be indirect. Tejada et al. (2011) reported that animal protein hydrolysate biostimulants stimulate activity of soil microorganisms, which in turn can induce systemic resistance immunity mediated by JAs from below ground organs to above ground parts, triggering JAs signaling in the whole plant (Jung et al., 2012).

### Pepton Improved Antioxidant Protection Under Water Stress

**FIGURE 5:**
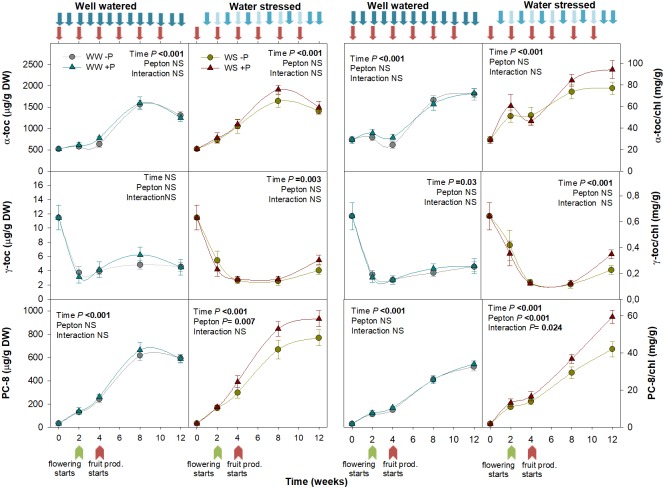
Variations in the endogenous content of tocochromanols in Pepton-treated tomato plants compared to controls under well-watered or water-stressed conditions. Data are the means ± SE of *n* = 10 individuals. Results of statistics (two-way ANOVA with Tukey HSD *post hoc* test) are shown in the inlets. Arrows represent days of watering and Pepton application. α-toc, α-tocopherol; γ-toc, γ-tocopherol; DW, dry weight; PC-8, plastachromanol-8.

Pepton significantly affected tocochromanol dynamics under water stress conditions. In particular, we found this biostimulant impacted the plastochromanol-8 content and plastochromanol-8/chlorophyll ratio (*P* = 0.007 and *P* < 0.001, respectively; Figure 5). While tocopherols are ubiquitous in photosynthetic tissues of all plant species, plastochromanol-8 and tocotrienols distribution is more limited. Both tocopherols and plastochromanol-8, but not tocotrienols, were found in tomato leaves, which is in agreement with previous studies (Kruk et al., 2014). Higher contents of plastochromanol-8 by the end of our study were probably due to increasing ambient temperature as the season progressed. Plastochromanol-8 and tocopherols have been shown to provide stress tolerance (Loyola et al., 2012; Fleta-Soriano and Munné-Bosch, 2017). Limited evidence of improved tocochromanol contents using biostimulants have been reported. Zhang and Schmidt (2000) observed enhanced α-tocopherol contents using hormone containing products and humic substances. In our study, the significant effect of Pepton on higher plastochromanol-8 content indicates that Pepton improves the antioxidant capacity of tomato plants. Interestingly, the higher plastochromanol-8 content was observed in parallel with higher JA content in Pepton-treated plants exposed to water stress. Since jasmonates can increase the expression of the gene encoding tyrosine aminotransferase (Sandorf and Holländer-Czytko, 2002), which is involved in the biosynthesis of homogentisate, it is likely that Pepton-related effects on jasmonates may exert a positive effect on platochromanol-8 accumulation. Additionally, the content of tyrosine (1.92%) in Pepton can partially explain the increase observed in tococromanols.

**FIGURE 6:**
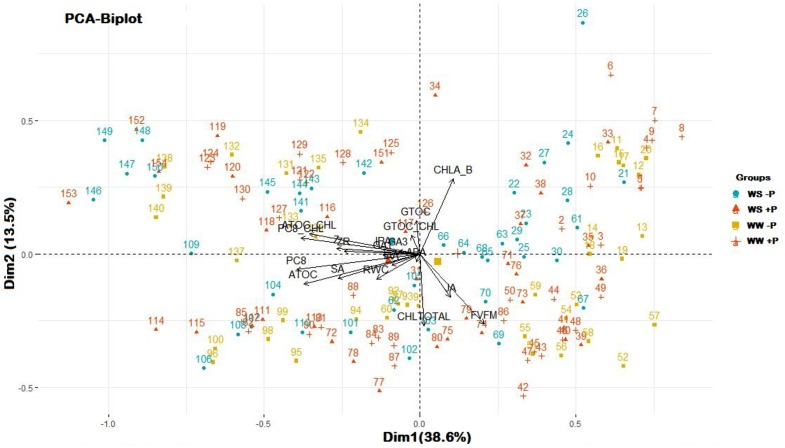
Principal Component Analysis of all variables measured in the study. Numbers represent each observation used to perform the analysis (*n* = 157) and symbols with colors represent each treatment (WS, water stressed; WW, well-watered; –P, without Pepton application; +P, with Pepton application, as shown in the legend). Symbols without number are the coordinates for a given group, calculated as the mean coordinates of the individuals in the group. Arrows represent each variable measured in the study. Dim1, principal component 1; Dim2, principal component 2. Percentages indicate to what extent the component explains observed variability. ATOC, α-tocopherol; ATOC_CHL, α-tocopherol/chlorophyll; CHLA_B, chlorophyll *a*/chlorophyll *b* ratio; CHLTOTAL, chlorophyll *a* + chlorophyll *b;* GTOC, γ-tocopherol; GTOC_CHL, γ-tocopherol/chlorophyll; PC8, plastochromanol-8; PC8_CHL, plastocromanol-8/chlorophyll.

A PCA was performed to unravel the main component explaining variability in this study (Figure 6). Principal component 1 (Dim1) captured 38.6% of the variance observed with plastochromanol-8, expressed as per unit of chlorophyll or per dry weight, as the greatest variable (17.52 and 16.39% of Dim1, respectively), followed by α-tocopherol and α-tocopherol/chlorophyll ratio. Observations were scattered through principal component 2 (Dim 2), which explained 13.5% of the whole variance, driven by the chlorophyll *a*/*b* ratio (25.19% of Dim2), chlorophylls (23.22% of Dim2) and *F*_v_/*F*_m_ ratio (22.05% of Dim2), but no clustering produced by treatments was observed. Even though observations were not clustered among treatments, they separated coordinate means in two groups through Dim1, WW, and WS, suggesting that factor “irrigation” may drive main variables involved in Dim1, which is in accordance with their response in stressful conditions. Antioxidants and chlorophyll related variables show opposite directions (Loyola et al., 2012; Cao et al., 2015; Fleta-Soriano and Munné-Bosch, 2017).

## Conclusion

**FIGURE 7:**
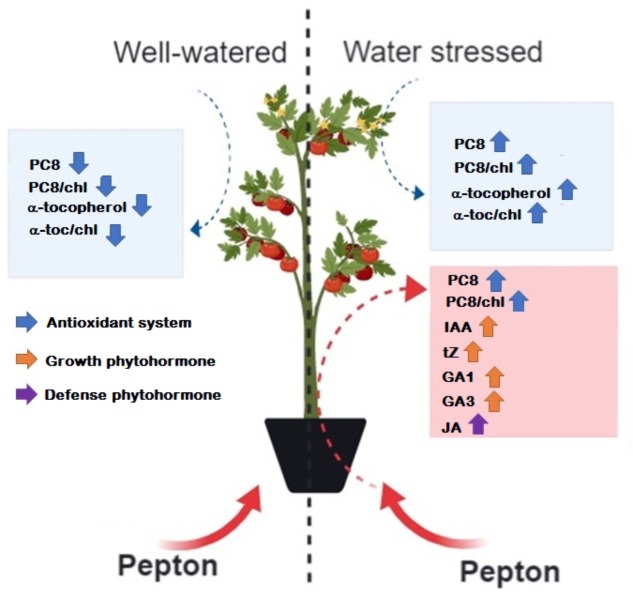
Schematic view of Pepton effects on tomato plants under water stress conditions. Information provided by PCA and ANOVA tests were considered. Blue boxes include main water stress effects on the variables studied as indicated by PCA and red boxes indicate Pepton effects on the variables studied as indicated by ANOVA tests. α-toc, α-tocopherol; chl, chlorophyll; PC8, plastochromanol-8. This figure was made with the help of web-based program BioRender (BioRender, Toronto, Canada).

It is concluded from the results obtained in this study that the enzymatically hydrolyzed animal protein-based biostimulant (Pepton) exerts a positive effect on the hormonal profile of tomato leaves and enhances abiotic defenses under water stress conditions, including defense-related phytohormones and antioxidants (a summary is depicted in Figure 7). Additional research is needed to more fully understand the mechanisms of action of hydrolyzed animal protein-based biostimulants to provide more accurate guidelines for its application in horticultural and agroecosystems.

## Author Contributions

JP and SM-B conceived and designed the experiments with the help of AC. AC wrote the manuscript with the help of JP and SM-B. AC prepared all the figures. All authors contributed to the discussion of ideas, revised, and approved the final manuscript.

## Conflict of Interest Statement

JP is employed by APC Europe S.L., Granollers, Spain. The remaining authors declare that the research was conducted in the absence of any commercial or financial relationships that could be construed as a potential conflict of interest.

## Abbreviations

ABA: abscisic acid
CKs: cytokinins
DW: dry weight
*F*_v_/*F*_m_: photosystem II maximum quantum yield efficiency
GAs: gibberellins
IAA: indole-3-acetic acid
IPA: isopentenyladenosine
JA: jasmonic acid
RWC: relative water content
SA: salicylic acid
*t*Z: *trans*-zeatin
*t*ZR: *trans*-zeatin riboside.
