# Supplementary material for: Hormonal Effects of an Enzymatically Hydrolyzed Animal Protein-Based Biostimulant (Pepton) in Water-Stressed Tomato Plants
Source: Front Plant Sci. 2019 Jun 12;10:758. doi: 10.3389/fpls.2019.00758 (PMC6582703; doi:10.3389/fpls.2019.00758)

Supplementary Figure 1. Climatic conditions in greenhouse during the study. Values are day mean of temperature (ºC) and relative humidity (%) of measures recorded every 20 minutes.

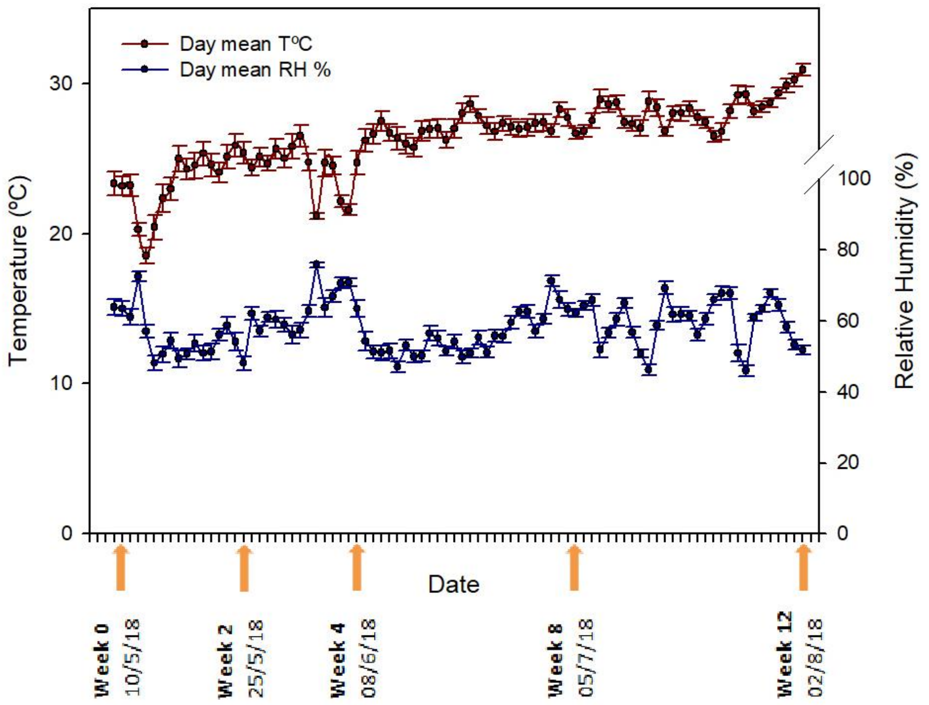

Supplement: Supplementary file 1 [file Image_1.pdf]
