# Supplementary material for: Hormonal Effects of an Enzymatically Hydrolyzed Animal Protein-Based Biostimulant (Pepton) in Water-Stressed Tomato Plants
Source: Front Plant Sci. 2019 Jun 12;10:758. doi: 10.3389/fpls.2019.00758 (PMC6582703; doi:10.3389/fpls.2019.00758)

Supplementary Figure 2. Phenotype of plants. Mild water deficit in water-stressed plants caused reductions in growth, which was less apparent in Pepton-treated plants.

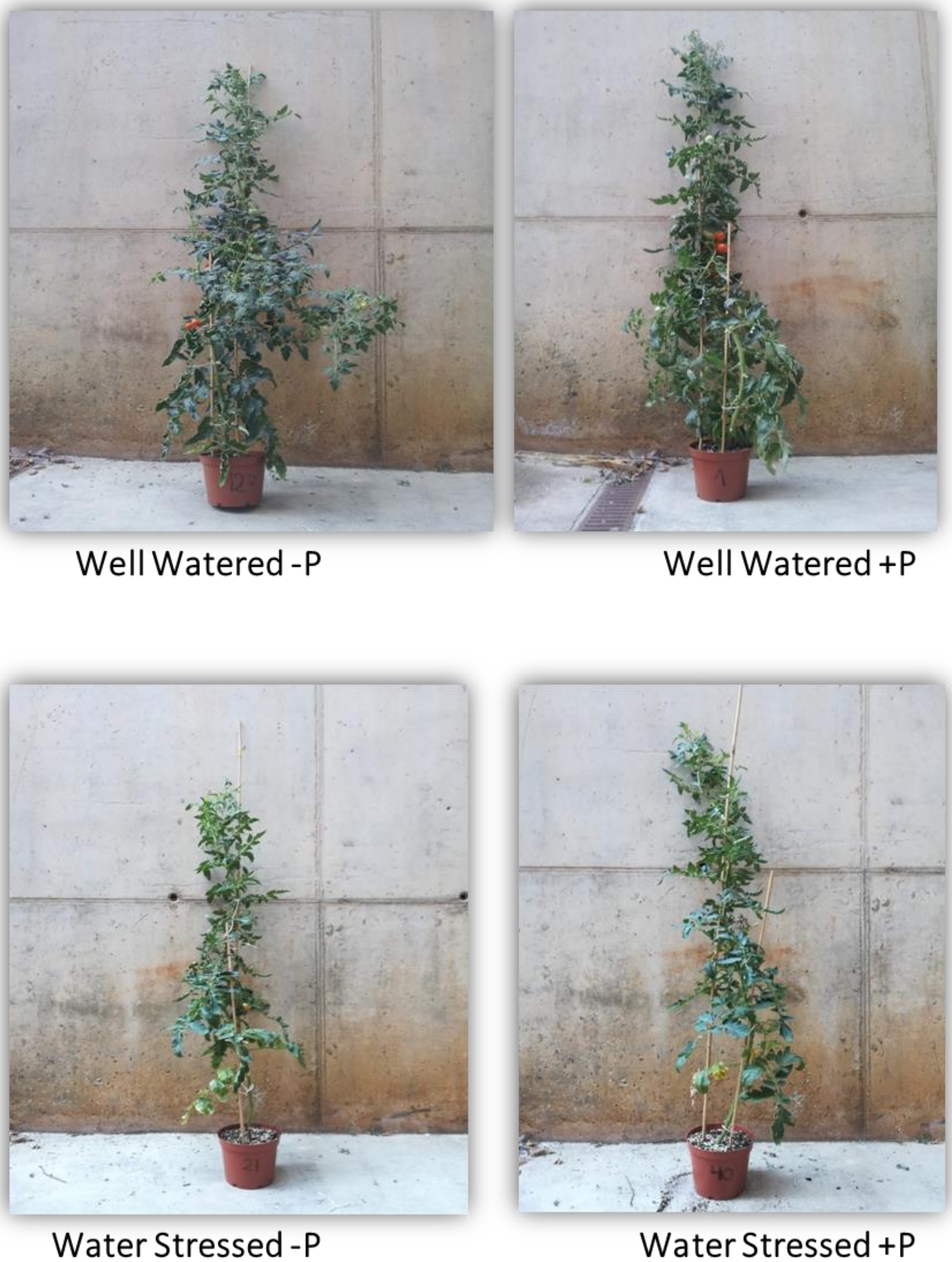

Supplement: Supplementary file 2 [file Image_2.pdf]
