# Supplementary material for: Hormonal Effects of an Enzymatically Hydrolyzed Animal Protein-Based Biostimulant (Pepton) in Water-Stressed Tomato Plants
Source: Front Plant Sci. 2019 Jun 12;10:758. doi: 10.3389/fpls.2019.00758 (PMC6582703; doi:10.3389/fpls.2019.00758)
Supplement: Supplementary file 3 [file Table_1.pdf]

Supplementary Table 1. Typical chemical and amino acid composition of Pepton as reported by the manufacturer.

	**Pepton**
Chemical composition	
Total organic matter, %	79.0
Total nitrogen, %	13.0
Organic nitrogen, %	12.0
Ammonia nitrogen, %	1.0
Ratio Carbon/Nitrogen	3.8
Potassium oxide (K2O), %	4.0
Phosphorous (P2O5), %	0.3
Calcium, ppm	300
Magnesium, ppm	500
Iron, ppm	3000
Amino acid composition	
Alanine, %	6.90
Arginine, %	3.22
Aspartic acid, %	9.93
Cysteine	<0.1
Glutamic acid, %	7.25
Glycine, %	4.06
Histidine, %	6.34
Isoleucine, %	0.15
Leucine, %	10.99
Lysine, %	7.19
Methionine; %	0.71
Phenylalanine, %	5.93
Proline, %	2.84
Serine, %	3.88
Threonine, %	2.47
Tryptophan, %	1.25
Tyrosine, %	1.92
Valine, %	6.79
Total amino acids, %	84.83
Free amino acids, %	16.52
